# PHYMYCO-DB: A Curated Database for Analyses of Fungal Diversity and Evolution

**DOI:** 10.1371/journal.pone.0043117

**Published:** 2012-09-13

**Authors:** Stéphane Mahé, Marie Duhamel, Thomas Le Calvez, Laetitia Guillot, Ludmila Sarbu, Anthony Bretaudeau, Olivier Collin, Alexis Dufresne, E. Toby Kiers, Philippe Vandenkoornhuyse

**Affiliations:** 1 Université de Rennes I, CNRS, UMR 6553 ECOBIO, Campus de Beaulieu, Rennes, France; 2 Université Européenne de Bretagne, Rennes, France; 3 Department of Ecological Science, Vrije Universiteit, Amsterdam, The Netherlands; 4 Université de Rennes I, CNRS, UMR 6074 IRISA, Campus de Beaulieu, Rennes, France; 5 Centre Scientifique et Technique du Bâtiment, AQUASIM, Nantes, France; Biodiversity Insitute of Ontario - University of Guelph, Canada

## Abstract

**Background:**

In environmental sequencing studies, fungi can be identified based on nucleic acid sequences, using either highly variable sequences as species barcodes or conserved sequences containing a high-quality phylogenetic signal. For the latter, identification relies on phylogenetic analyses and the adoption of the phylogenetic species concept.

Such analysis requires that the reference sequences are well identified and deposited in public-access databases. However, many entries in the public sequence databases are problematic in terms of quality and reliability and these data require screening to ensure correct phylogenetic interpretation.

**Methods and Principal Findings:**

To facilitate phylogenetic inferences and phylogenetic assignment, we introduce a fungal sequence database. The database PHYMYCO-DB comprises fungal sequences from GenBank that have been filtered to satisfy stringent sequence quality criteria. For the first release, two widely used molecular taxonomic markers were chosen: the nuclear SSU rRNA and EF1-α gene sequences. Following the automatic extraction and filtration, a manual curation is performed to remove problematic sequences while preserving relevant sequences useful for phylogenetic studies. As a result of curation, ∼20% of the automatically filtered sequences have been removed from the database. To demonstrate how PHYMYCO-DB can be employed, we test a set of environmental Chytridiomycota sequences obtained from deep sea samples.

**Conclusion:**

PHYMYCO-DB offers the tools necessary to: (i) extract high quality fungal sequences for each of the 5 fungal phyla, at all taxonomic levels, (ii) extract already performed alignments, to act as ‘reference alignments’, (iii) launch alignments of personal sequences along with stored data. A total of 9120 SSU rRNA and 672 EF1-α high-quality fungal sequences are now available.

The PHYMYCO-DB is accessible through the URL http://phymycodb.genouest.org/.

## Introduction

In recent years there has been an exponential increase in the number of gene sequences available in public-access databases. This is the result of new developments in molecular techniques and new generation sequencers that allow the collection of data at great speed. The use of molecular taxonomic markers associated with phylogenetic analyses has revealed considerable genetic diversity in fungi, especially those that are cryptic, unculturable or not easily distinguishable by morphological characters (e.g. [1]). As the species concept is employed for diversity measurements, systematics and evolutionary analyses [2], an efficient means of identifying boundaries, and thus number of species, is required. Molecular methods and the implicit adoption of the phylogenetic species concept [3] offer a standardized approach to delimit groups of organisms (e.g. [4]–[6]). Thanks to progress in sequencing technologies and bioinformatic methods, the detection of orthologous sequences using databases is relatively efficient. This approach can also be successfully applied to organisms that are not available in culture, increasing our ability to identify new diversity in various habitats [7], [8]. Of course, this approach requires choosing a relevant molecular marker which: (i) targets a nucleic acid sequence with a limited proportion of homoplasy (i.e. correspondence between parts arising from evolutionary convergence), (ii) contains high phylogenetic information which is not sensitive to paralogy (i.e. single copy genes or highly conserved genes). This allows for accurate characterization of evolutionary affinities.

In this context, the nuclear gene coding for the small subunit of the ribosomal RNA (SSU rRNA) is often seen as the ‘ultimate’ molecular marker [9], (for review [10]). The SSU rRNA gene is present in all living organisms. Its sequence is highly conserved between taxa, reflecting strong functional constraints on the translational machinery. Indeed, most mutations in the SSU rRNA gene sequence reduce the stability of the secondary structure of the SSU rRNA molecule and thus the efficiency of protein synthesis. Furthermore, this gene, like other informational genes, appears to be less subject to horizontal gene transfers and is believed to provide better inferences of ‘true’ phylogenies [11]. Although the SSU rRNA gene can have a multicopy status within a single fungal genome, sequence variations have been shown to be extremely low or null. For example, from available complete annotated genomes (http://www.genomesonline.org/cgi-bin/GOLD/index.cgi), *Saccharomyces cerevisiae* has two SSU rRNA copies both on its chromosome XII. *Encephalitozoon cuniculi*, a Microsporidia, has two SSU rRNA genes copies one on its chromosome I, the other on chromosome IV. In these two cases, the copies display 100% identity. This is not surprising since the SSU rRNA gene is highly conserved. Thus this gene is less sensitive to paralogy compared to LSUrRNA gene and ITS where variations among copies have been clearly shown (e.g. [12]–[14]).

A second advantage of using the SSU rRNA gene sequence is its huge representation in international public databases - GenBank [15], EMBL/ENA [16], DDBJ [17] – which facilitates comparisons between a wide variety of organisms (for review [18]). One disadvantage is that because the SSU rRNA gene is highly conserved, the resolution of the phylogenetic analyses is poor for youngest fungal groups within Ascomycota. Other genes, such as those encoding for the elongation factor EF1-α (*tef1*), for β-tubulin (*tub1*, *tub2*), actin (*act1*), or for RNA polymerase II subunits (*rpb1* and *rpb2*), can be used as alternative markers. Among these ones, EF1-α sequence data are the most abundant but only represent a small fraction of the amount of SSU rRNA yet available (i.e. less than 7% of the total number of sequences contained in PHYMYCO-DB). Generally present as a single copy gene, the EF1-α gene is involved in protein synthesis and displays a higher mutation rate than SSU rRNA gene. Because of these attributes, EF1-α protein sequences have been used to resolve phylogenetic affinities between eukaryotic organisms [19]–[21], and particularly the sister clade relationship of animals and fungi [22]. The gene sequences also have the potential to help resolve phylogenetic relationships between closely related fungi [21], [23]–[24], but they contain a higher proportion of homoplasious positions compared to SSU rRNA gene sequences. Studies of both SSU rRNA genes and EF1-α genes could greatly improve the resolution of fungal phylogenetic affinities. An online database incorporating data from both these sequences is a key step to achieving improved phylogenetic resolution for fungi.

### Pollution of public sequence database and the aim of PHYMYCO-DB

One major obstacle for international public databases is constant pollution by non-negligible proportions of compromised sequences (GenBank/EMBL/DDBJ). This problem, discussed in several articles and journal forums (e.g. [25]–[32]), is becoming more and more obvious, but solutions remain elusive. Problematic data can arise from many different origins, including: (i) erroneous specimen identification [27], (ii) the use of separate names for different sexual stages [30], (iii) differences in taxonomy among specialists [27] and/or advances in knowledge since the time the sequence was deposited leading to wrong designations [29], (iv) the lack of precision in the description of the deposited sequences making their interpretation difficult [33], (v) sequences resulting from artefactual origin (i.e. chimeric sequences), and (vi) sequences of poor quality with undefined positions. Even more problematic is the erroneous annotated sequences that propagate within open access databases because of phylogenetic misinterpretation. Additionally, more and more sequence assignments are based solely on identity searches using heuristic local alignment (i.e. BLASTn searches). All these mistakes have the potential to jeopardize interpretations. Therefore, assessing the reliability of sequences is an increasingly important prerequisite to analyses.

Many of these errors can be limited via expert curation. Expert curation is critical for the continued advancement of the field because it allows for the production of sequence databases, containing accurate and reliable sequences. To date, most curated databases specialize in particular taxonomic groups (e.g. [34]), collect data associated to each nucleic acid sequence, and work with specimens validated by experts and deposited in public reference collections (e.g. [33]). Several important tools, such as the Ribosomal Database Project [35], SILVA [36], Greengenes database [37] exist online for the analysis of SSU rRNA gene sequences. Apart from SILVA, these databases use automated filters to remove part of the polluting sequences. However, manual curation is an essential component of these projects and should aim to be even more stringent.

Based on lessons learned from other curated databases, our aims at PHYMCO-DB are to: (i) develop an easy-to-use fungal-dedicated database with stored sequences of high quality, (ii) use selected molecular markers that are widely acknowledged, namely SSU rRNA and EF1-α, (iii) produce a tool, based on anchor sequences covering the fungal tree, that can be automatically updated, along with an expert curation of the new sequences, (iv) produce high quality multiple alignments for use in testing environmental sequences or evolutionary hypotheses.

## Database Structure : Design and Implementation

**Figure 1:**
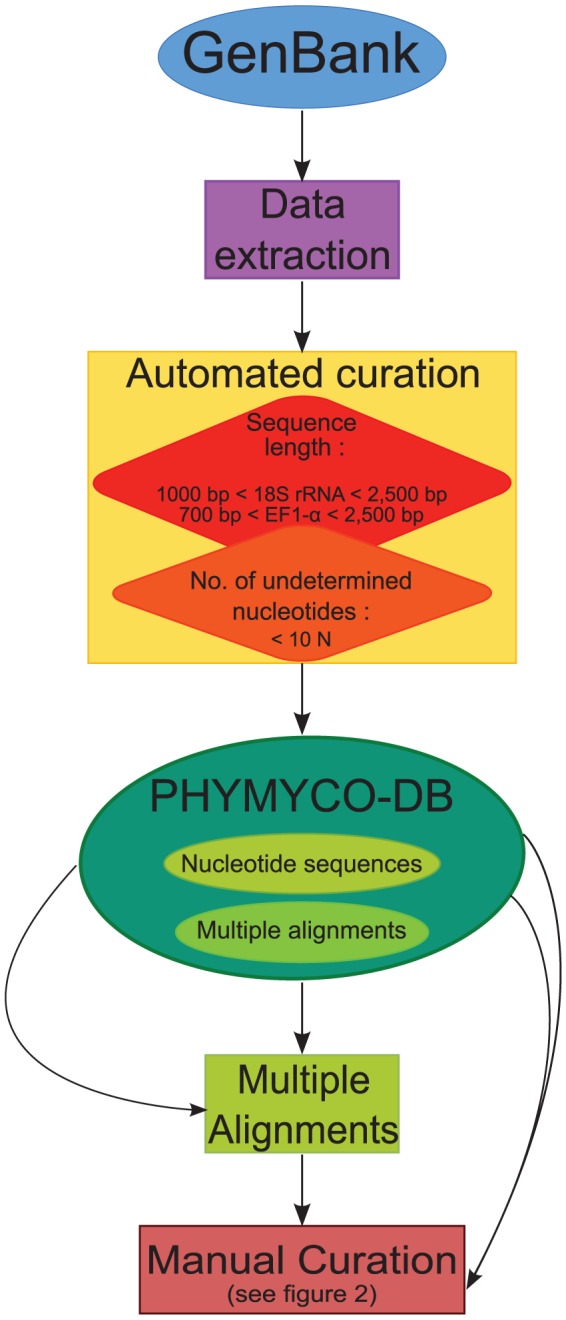
Flowchart of the data in the PHYMYCO-DB. The arrows indicate the flow of gene sequences extracted from the GenBank database, through the automated and manual curation steps. All the sequences made available to users has passed the 2 curation processes. After each upgrade of the database (i.e. 4 times per year), expert manual curation is performed.

The sequences constituting PHYMYCO-DB version 1 (Fig. 1) were retrieved in October 2011 from the release 185 of GenBank (NCBI). The nuclear SSU rRNA and EF1-α genes sequences are extracted from the GenBank database, using the following queries: “[organism] and (ssu|SSUrRNA|SSU rRNA|18SrRNA|18S|) not (16S|mitoch*|28S|5.8S|ITS|Internal Transcribed Spacer|internal transcribed spacer|)” and “[Organism] and (EF1 alpha|EF-1 alpha|EF1-alpha|EF-1alpha|EF-1-alpha|EF1alpha|EF1a|)”. After this extraction step, automatic quality filter parameters are applied. For SSU rRNA, nucleic acid sequences that are shorter than 1000 nucleotides and longer than 2500 nucleotides are rejected. Likewise for EF1-α genes, sequences shorter than 700 nucleotides and longer than 2500 nucleotides are discarded. Also sequences containing more than 10 consecutive undetermined nucleotides are excluded. According to the automatic quality criteria, all accepted sequences are then stored in a MySQL 5 relational database. The MySQL table structure is presented as a figure available in supplementary online information (Fig. S1). PHYMYCO-DB is automatically updated 4 times a year and is managed by administrators using the web interfaces developed with PHP version 4 programming language.

**Figure 2:**
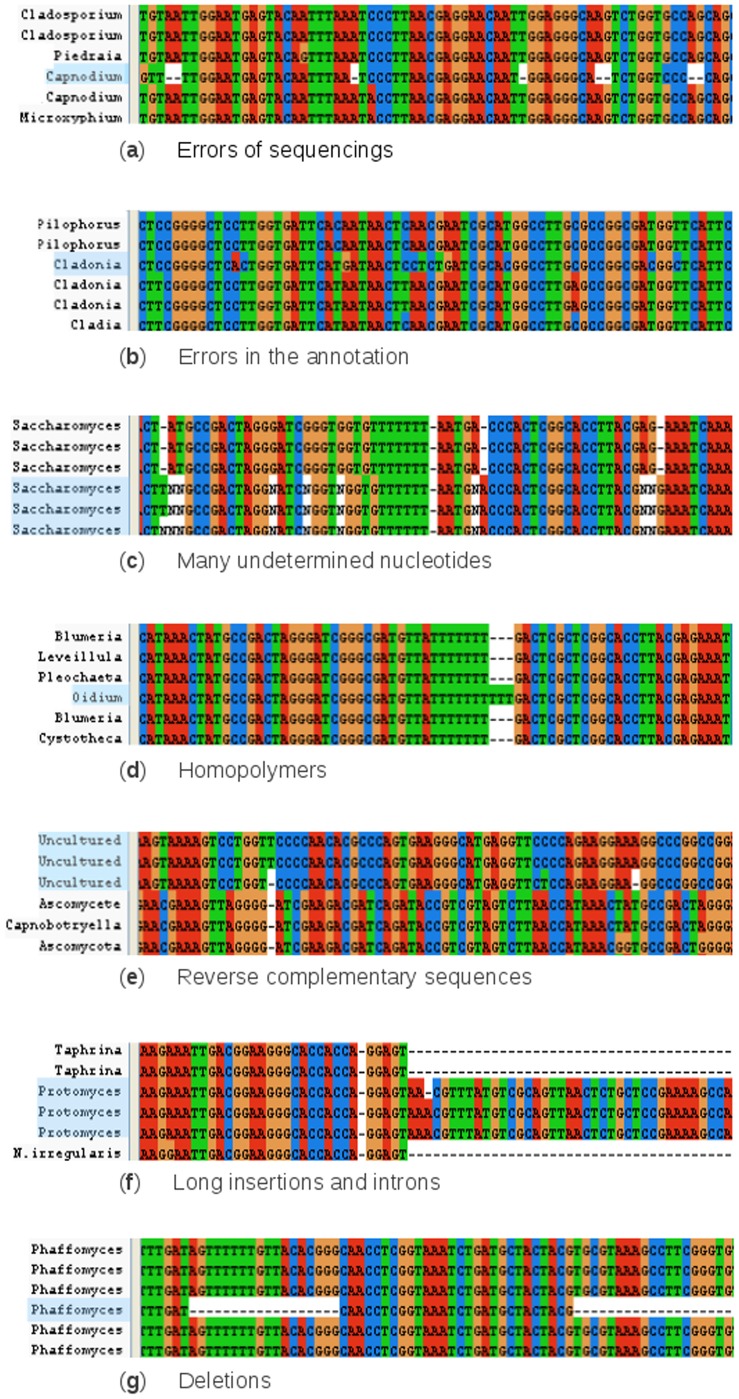
Visualisation of sequences deleted by the manual curation after alignment (ClustalX 2.1). The sequences highlighted in blue illustrate examples of sequences removed from PHYMYCO-DB. The compromised nature can stem from erroneous sequencing (e.g. repeated gaps), wrong annotation (e.g. sequence corresponding to another clade), high numbers of undetermined nucleotides, homopolymers insertions, erroneous alignment or reverse complementary sequences and presence of long insertions and introns or presence of deletions.

Following automatic filtering, datasets are then cross-checked by expert curators (hereafter ‘expert curation’). Multiple alignments are performed using Clustal X 2.1 [38] on small sequence groups (<400 sequences), which are closely related to obtain a high-quality alignment and to make the expert curation as accurate as possible. Sequences are deleted from the alignment and from the database in a manual cleaning process if they contain: errors of sequencing (i.e. containing several substitutions that are not found anywhere else, Fig. 2), errors in the annotation (i.e. a sequence with a naming inside a different group, Fig. 2), homopolymers insertions (Fig. 2), many undetermined nucleotides (Fig. 2), erroneous alignment or reverse complementary sequences (Fig. 2). This expert curation is time consuming but essential to obtain reliable sequences and high-quality alignments. By adopting strict rules of expert curation, subjectivity and mistakes become minimal. Following expert curation, species redundancy (i.e. identical sequences) are retained in the database to keep sequences arising from different origin and ecological settings. The detection of dubious sequences from the alignments does not result in correction of the sequence in international databases. They are, however, all removed from PHYMYCO-DB. When corrections are made for a given sequence, a new registration number is provided by GenBank for example. In this case, the corrected sequence will be automatically extracted (i.e. 4 updates per year) and will be examined by one of the expert curators.

During our development process, it became clear that our automatic filters were not stringent enough to retrieve only trustworthy sequences. For example, SSU rRNA can present intron-like regions which could also be chimeric insertions. Introns are abundant in particular lineages of fungi, especially within lichen-forming fungi (Ascomycota). These fungi can display up to eight introns in the SSU rRNA gene, as for example found in the taxon *Physconia* [39]. At the expert curation stage, we noticed that the position of introns was not consistently given in the deposited sequence description, and they were detectable after the alignment only. When a sequence containing non-positioned introns was the only sequence of a particular genus, this sequence was kept, Otherwise the sequence was discarded from PHYMYCO-DB. Employing our curation principles, we discarded 2090 additional unreliable sequences, i.e. 18% of the sequences extracted from GenBank.

Following the curation steps, 8757 SSU rRNA gene sequences have been stored in PHYMYCO-DB (5088 Ascomycota, 2088 Basidiomycota, 366 Chytridiomycota, 1046 Glomeromycota, and 532 Zygomycota). PHYMYCO-DB also contains 648 EF1-α gene sequences (294 Ascomycota, 189 Basidiomycota, 10 Chytridiomycota, 25 Glomeromycota, and 154 Zygomycota). Our database contains less fungal sequences than SYLVA because of the level of curation stringency. All fungal genera has at a minimum one representative sequence within PHYMYCO-DB. Because of the heterogeneity among the number of sequences per taxonomic rank, and because we wanted a limited number of sequences for each alignment, the taxonomic level within these alignments is variable (family to phylum level). We therefore produced a total of about 50 ‘reference’ alignment files. These online alignments contain mainly full-length sequences, even if rare, very long sequences were cut at the same length as the others. This was done to keep maximum information available. This is especially useful for designing primers, and to give a greater freedom for manipulation by online users.

## Tools within PHYMYCO-DB

We designed PHYMYCO-DB with specific tools to facilitate online use. Firstly, users can easily select sequences by browsing our interface through hierarchical taxonomic lineages presented in an arborescent structure (GenBank taxonomy), and then download them in a FASTA format file. The number of sequences stored in the database for each taxonomic level is given in brackets. Secondly, users can download an alignment file using a filter to find an alignment with the gene and the taxonomic rank requested. Special attention must be paid to the fact that some sequence characteristics in PHYMYCO-DB format are inherited from the extraction of GenBank sequences. For example, in some cases (e.g. Agaromycotina, a subphylum of Ascomycota), information on sequences taxonomy was associated to a ‘no rank’ tag in GenBank. To avoid the problem that these sequences are mistakenly placed in another taxonomic group, they were qualified as ‘undefined’ at the subphylum rank in PHYMYCO-DB. For the next lower taxonomic rank, no known tag problem exists. Environmental sequences have, by definition, no clear taxonomic ranking. Therefore, they were also qualified as ‘undefined’, but only until the lowest taxonomic rank. These are important features to take into account when using the PHYMYCO-DB.

Thirdly, users can launch a ClustalW 2.0 alignment on our back-end computer clusters by uploading their own personal sequences in a FASTA or ALN format file. A future PHYMYCO-DB release will offer the possibility to select the multiple alignment tool (i.e. ClustalW, MUSCLE, and MAFFT). Currently, users can choose to append an outgroup or sequences from a particular PHYMYCO-DB taxonomic group. We anticipate that this tool will be very efficient when combined with phylogenetic analyses for investigating the sequence diversity of fungal amplicons from an environmental sample and even to identify new fungal lineages.

## PHYMYCO-DB as a Tool for Phylogenetic Identifications and Inferences

Based on a well-developed theoretical corpus, phylogenies can be computed using several different approaches (e.g. [40]). From a mathematical point of view, the maximum likelihood phylogenetic reconstruction provides the best possible tree for a given explicit sequence evolution model. The model that best fits the aligned sequence data can be selected, after using the popular Modeltest [41]. Achieving a good alignment is therefore of tremendous importance for good interpretation. Alignments should be refined using an ‘influence function’ that allows the removal of outlier columns from the matrix (i.e. nucleotide position where the phylogenetic signal differs from the general phylogenetic information recorded in the dataset) [42]. This approach allows for a ‘blind detection’ of outliers using measures of each site in a context of a ML phylogenetic reconstruction. It must be emphasized that the sequence-based identification using SSU rRNA gene could be at the species level or at higher taxonomic levels depending on the fungal affiliation.

**Figure 3:**
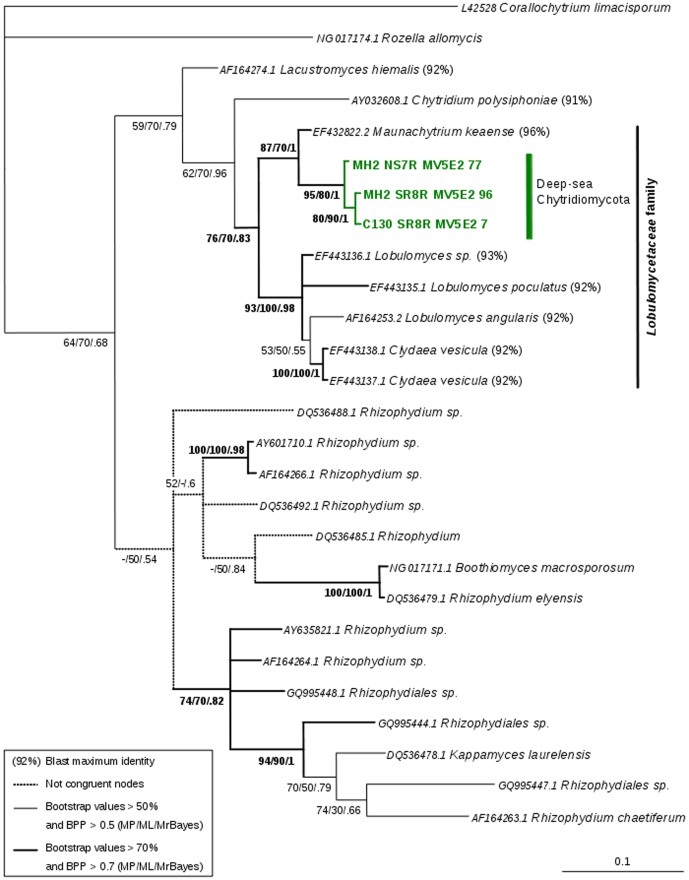
SSU rRNA phylogenetic positions of deep-sea Chytridiomycota (colored terminals) along with the closest known related SSU rRNA fungal sequences. Topology was built using MrBayes v.3.1.2 (Scale bar: 0.1 estimated substitutions per site, 3000000 generations sampled every 100 generations and an average standart deviation of split frequencies of 0.004140) from a ClustalW 2.1 alignment. The model GTR+I+G was designated by jModelTest 0.1. Node support values are given in the following order: Maximum Parsimony/Maximum Likelihood (both calculated with PAUP 4.0β10 version, 500 bootstraps)/MrBayes. *Corallochytrium limacisporum* (L42528), a putative choanoflagellate, was used as outgroup. *Maunachytrium keaense* (it is not part of PHYMYCO-DB) was also used to help build the tree. All sequences are listed with their GenBank accession numbers. The topologies were congruent apart from doted lines indicated in the figure. Thin lines show bootstrap values >50% and BPP >0.5 (MP/ML/MrBayes) and thick lines: bootstrap values >70% and BPP >0.7 (MP/ML/MrBayes). The sequences belonging to the *Lobulomycetaceae* family are indicated with their BLASTn percentage of maximum identity compared to the three deep-sea Chytridiomycota OTUs.

Following the above strategy, we provide an analysis of chytrid diversity as a proof of concept. Sequencing of the SSU rRNA gene was achieved by targeting chytrids from deep marine hydrothermal samples (ciPCR). First, the alignment of SSU rRNA gene sequences of the Chytridiomycota from PHYMYCO-DB were used to design specific primers manually. Two sets of designed primers covered the V3 and V4 variable regions and were suitable for pyrosequencing: C130 (5′TACCTTACTACTTGGATAACCG3′) with SR8R (5′TCAAAGTAAAAGTCCTGGATC3′) modified from Vilgalys lab webpage (http://www.biology.duke.edu/fungi/mycolab/primers.htm), and MH2 (5′TTCGATGGTAGGATAGAGG3′) [43] with SR8R. Another set of primers, expected to be universal for fungi and to produce longer amplicons, were also tested: MH2 with NS7R (5′ATCACAGACCTGTTATTGCC3′) modified from [44]. Primers specificity was checked with a sample from a hydrothermal site from which several sequences of chytrids were retrieved [45]. The resulting sequences (GenBank accession numbers JN986721 to JN986723) were analyzed using the corresponding ‘reference’ alignment in PHYMYCO-DB and the sequences having the highest similarity score in BLASTn. The computed phylogeny highlights the presence of a new group within the Chytridiomycota phylum (Fig. 3). The three OTUs present high identity level (>98%) with environmental sequences ,and form a monophyletic group whose closest described relative is a sequence from the genus *Maunachytrium*. These OTUs constitute a new clade in the Lobulomycetaceae family [46]. BLASTn searches of these environmental sequences return the *Maunachytrium* sequence as the best hit, with a maximal identity of 96%. The widely used BLAST-based annotation for environmental sequences, would end with an assignation to *Maunachytrium keaense* or *Maunachytrium* sp. However, by choosing a phylogenetic approach, the analysis goes into greater depth. The initial positioning of these sequences suggests that they form a new clade within the Lobulomycetaceae family, outside the *Maunachytrium*, *Lobulomyces* (maximal identity 93%) and *Clydaea* (maximal identity 92%) genera.

This exercise thus highlights important differences between phylogeneticaly based annotation and BLASTn annotation. More and more identifications rely solely on BLAST searches which allow for faster analyses of the rapidly increasing numbers of environmental sequences. Indeed many analyses and tools developed for mass sequencing are based on BLAST searches (e.g. MEGAN). We would argue that this approach is less conservative and more prone to mistakes. The use of phylogenetic approaches, when it is possible should be favoured, to avoid increasing the presence of polluting sequences in international sequences databases.

## Discussion

The release of PHYMYCO-DB is expected to provide comprehensive access to fungal sequences for two phylogenetic markers (SSU rRNA and EF1-α genes) obtained from cultivated isolates, as well as environmental samples. As a result of deep sequence cleaning, the aligned sequences available in PHYMYCO-DB are of high quality (Fig. 1). To our knowledge, this curation strategy provides a novel approach to the problem of database pollution. As such, we anticipate that it will complement other existing databases such as the “Assembling the Fungal Tree Of Life” project (AFTOL) [47], UNITE [33], [48] and MaarjAM [34] which are restricted to fungal sequences.

Curation and annotation of ITS is made possible through the web-based-workbench of PlutoF [49]. Initially, the UNITE system contained ITS and nLSU/28S rRNA gene sequences from Basidiomycota and Ascomycota. Based on recent work, the ITS region is now being suggested as a possible universal DNA barcode marker for fungi [50]. It is accepted that the ITS region is valuable at species level and so, more taxonomically informative than SSU rRNA gene sequences for analysing groups of organisms that have emerged ‘recently’ and are closely related [51], e.g. Ascomycota and Basidiomycota. The ITS region is also often used to resolve phylogenetic relationships at the species level or at the infraspecific level [52]. However, as the ITS region displays high sequence variability, even within a given organism as in Glomeromycota (i.e. [13]), obtaining reliable alignments with this marker can be difficult [53] and potentially precludes multiple alignments. This is because accurate comparisons are hindered by the accumulated homoplasy and the high frequency of insertion/deletion events. The use of the SSU rRNA sequences is interesting since new groups, within all the fungal phyla including Ascomycota and Basidiomycota, can be detected (i.e. [1], [54]). The MaarjAM database has focused on SSU rRNA gene of arbuscular mycorrhizal fungi (Glomeromycota), with associated metadata. The existence of this database and the potential emergence of others should be encouraged. It enables the community to have access to reliable sequences.

For fungal sequence annotations and phylogenetic interpretations of fungal environmental sequences, one of the main advantages of PHYMYCO-DB is to facilitate the primer design and subsequent phylogenetic analyses of amplicons as shown in the example above (Fig. 3). The use of PHYMYCO-DB to perform expert analyses appears to be complementary to BLASTn, the latter allowing a quick look of the query sequence proximity compared to the available sequences. From the phylogenetic analyses performed one arising interpretation is that different apparent polyphyletic groups may be a consequence of wrong annotations. We anticipate that the use of PHYMYCO-DB will help to limit incorrect SSU rRNA and EF1-α genes fungal annotation propagation in sequence databases.

## Availability and Future Directions

The PHYMYCO-DB is available via a web-based interface at http://phymycodb.genouest.org/ on the GenOuest bioinformatics platform web site. The web interface is divided into 2 parts. The first part, entitled “*DB admin*”, is restricted to the administrators for use in cleaning and optimising the database. The second part, entitled “*DB explore*”, is publicly accessible to all users. The next set of PHYMYCO-DB releases will include (i) the provision of alignment files in which outlier nucleotides identified from influence functions [42] will be highlighted, so that users can then delete these sites (ii) taxonomic modifications within Chytridiomycota and Zygomycota after Hibbett et al. (2007) [55] and after Jones et al. (2011) [5]. PHYMYCO-DB will continue to expand with new genes. We are currently investigating β-tubulin (*tub1*, *tub2*), actin (*act1*), and RNA polymerase II subunits (*rpb1* and *rpb2*) as potential interesting targets. PHYMYCO-DB will also be improved by incorporating all the finished fungal genomes available, and increasing the diversity of tools to perform multiple alignments.

## Supporting Information

Figure S1 **MySQL table structure of PHYMYCO-DB.**
(TIF)
