# Supplementary figures and images for: PHYMYCO-DB: A Curated Database for Analyses of Fungal Diversity and Evolution
Source: PLoS One. 2012 Sep 13;7(9):e43117. doi: 10.1371/journal.pone.0043117 (PMC3441585; doi:10.1371/journal.pone.0043117)

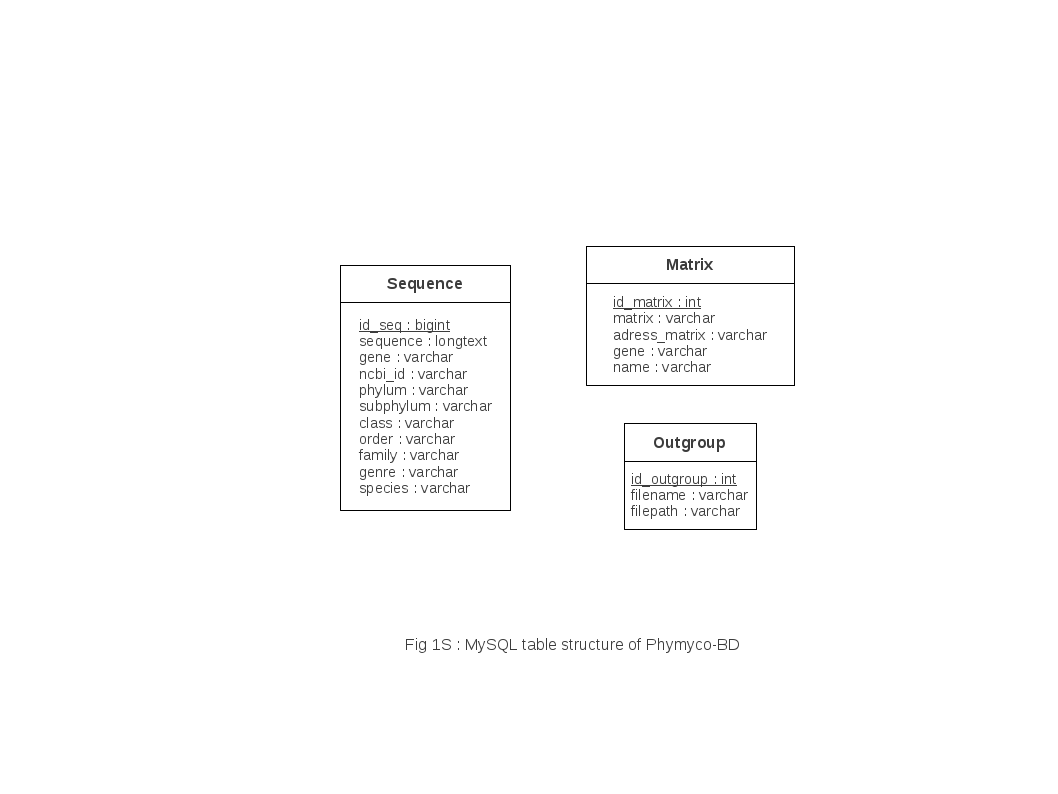

Supplement: Figure S1 — MySQL table structure of PHYMYCO-DB. (TIF) [file pone.0043117.s001.tif]
